# eHealth in Treatment of Offenders in Forensic Mental Health: A Review of the Current State

**DOI:** 10.3389/fpsyt.2018.00042

**Published:** 2018-02-21

**Authors:** Hanneke Kip, Yvonne H. A. Bouman, Saskia M. Kelders, Lisette J. E. W. C. van Gemert-Pijnen

**Affiliations:** ^1^Centre for eHealth and Wellbeing Research, Department of Psychology, Health and Technology, University of Twente, Enschede, Netherlands; ^2^Department of Research, Stichting Transfore, Deventer, Netherlands; ^3^Optentia Research Focus Area, North-West University, Vanderbijlpark, South Africa

**Keywords:** eHealth, interventions, technology, forensic psychiatry, offenders

## Abstract

**Background:**

Treatment of offenders in forensic mental health is complex. Often, these in- or outpatients have low treatment motivation, suffer from multiple disorders, and have poor literacy skills. eHealth may be able to improve treatment outcomes because of its potential to increase motivation and engagement, and it can overcome the predominant one-size-fits-all approach by being tailored to individual patients.

**Objective:**

To examine its potential, this systematic review studies the way that eHealth has been used and studied in forensic mental health and identifies accompanying advantages and disadvantages for both patients and treatment, including effectiveness.

**Methods:**

A systematic search in Scopus, PsycINFO, and Web of Science was performed up until December 2017. Studies were included if they focused on technological interventions to improve the treatment of forensic psychiatric patients.

**Results:**

The search resulted in 50 studies in which eHealth was used for treatment purposes. Multiple types of studies and technologies were identified, such as virtual reality, web-based interventions, and videoconferencing. The results confirmed the benefits of technology, for example, the acquisition of unique information about offenders, effectiveness, and tailoring to specific characteristics, but indicated that these are not fully taken advantage of.

**Discussion:**

To overcome the barriers and obtain the benefits, eHealth has to have a good fit with patients and the forensic psychiatric context. It has to be seamlessly integrated in existing care and should not be added as an isolated element. To bridge the gap between the current situation and eHealth’s potential, further research on development, implementation, and evaluation should be conducted.

## Introduction

Forensic mental health treatment focuses on the intersect between psychiatry and the law by dealing with the relationship between, assessment and treatment of mental illness and criminality of people whose behavior has led, or could lead, to offending (1, 2). Besides treatment of psychiatric disorders, a primary goal is to prevent criminal recidivism via addressing offense-related factors such as antisocial behavior or coping skills. These factors should be addressed via interventions and therapies based on evidence-based approaches such as cognitive behavior therapy (3) and the risk-needs-responsivity principles (4). However, developing and implementing such in-person interventions in this complex field has proven to be challenging. For example, meta analyzes on interventions targeting batterers, juvenile offenders, and relapse prevention of offenders found low overall effectiveness on clinical measures (5). These results indicate that there is room for the improvement in interventions in forensic mental health. A solution might be found in the use of eHealth in treatment. eHealth can be defined as technologies such as web-based interventions, apps, wearables, or virtual reality (VR), to improve and support health, well-being, and quality of care (6). Many studies acknowledge eHealth’s added value for general mental health [e.g. references (7–9)] but it is not yet clear what its advantages for forensic mental health treatment could be.

There are multiple complicating factors within forensic mental health that can influence the success of existing in-person interventions. Among other things, the forensic psychiatric population has specific characteristics, such as the low motivation that forensic psychiatric outpatients generally have for their, often mandated, treatment (10). Studies suggest that mandated treatment outcomes are often worse compared with patients without mandated treatment (11). Another complicating factor is that a large share of the forensic population displays psychiatric comorbidity (12, 13), and that not one, but multiple factors cause delinquent behavior and should be addressed in treatment (14). Furthermore, forensic psychiatric patients are often disproportionately poor, unemployed, and have lower literacy rates (15), which might affect their capability of being engaged in and adhering to interventions. Despite the complexity and diversity that the low motivation, low literacy, and comorbidity of forensic psychiatric patients bring to treatment, most interventions have a “one-size-fits-all approach.” Many interventions do not take individual differences into account (16) despite the acknowledgment of the importance of tailoring interventions to specific characteristics of individual patients (17–19). The use of eHealth technology within in-person treatments and interventions, which is referred to as blended care (20), could be a way to increase this required fit between interventions and patients. eHealth has several characteristics that could be of added value for forensic mental health.

First of all, the content, way of communicating and design of technology, can be tailored to subgroups or individual users, based on their characteristics, needs, or context (21). This tailoring or personalization of eHealth interventions creates a better fit between the technology and the individual user and consequently addresses the complexity and diversity of the forensic mental health domain. Tailoring has been proven to enhance user engagement and effectiveness of multiple eHealth interventions (22–25). The use of tailoring prevents the aforementioned one-size-fits all approach of interventions, which does not seem to suit the complex and broad forensic population (17).

Another way to account for the complexity of the target group is by the use of existing protocols, guidelines, and evidence-based theories in interventions, which is advised for forensic and also general mental health (26, 27). However, many existing in-person interventions for forensic mental health care are not theory-based, or treatment integrity by therapists is not always satisfactory (16, 28). Technology offers the possibility to deliver interventions based on theory and guidelines to patients in a standardized way to increase effectiveness, while still being able to tailor its content to individual patients. Consequently, eHealth can standardize care and interventions by incorporating existing guidelines (29).

eHealth has several characteristics that can increase the motivation of forensic psychiatric patients in managing their own care. If patients are motivated, they are more likely to have higher adherence to an intervention, meaning that they use it in the intended way and obtain positive treatment outcomes compared with disengaged and unmotivated patients (30, 31). Patient engagement can be achieved by using innovative, state-of-the-art technologies that appeal to the patient, like serious gaming, wearable technology, or VR (7, 30, 32). Many of these new technologies do not primarily rely on conscious cognitive reflection, but mainly create experiences, which suits the lower literacy and education of the average forensic psychiatric patient. The way an intervention is designed can contribute to adherence as well, for example, via the application of principles from persuasive design (33, 34). Finally, tailoring of a technology can positively impact adherence since it increases the fit with a patient’s needs and wishes and can increase the perceived personal relevance of an intervention (34), which has a positive influence on patient motivation.

Based on research on eHealth in general, many potential benefits of eHealth for forensic mental health can be identified. However, it is not yet clear in how far these advantages are relevant for and actualized in forensic mental health. To determine what the added value of eHealth is, this systematic review aims to provide an overview the current state of affairs of eHealth research in forensic mental health. This is accomplished by studying the types of studies, the studied technologies, and the mentioned advantages and disadvantages. Based on these findings, it can be identified if and how eHealth can have added value for forensic mental health, and domain-specific recommendations can be provided on how it can reach this potential added value. The research questions were generated via the PICOS method. The population was defined as forensic psychiatric patients, and interventions were all types of technologies used in the treatment of forensic psychiatric patients. Because of the explorative nature of this review, we did not address comparators in the research questions. Main outcomes were types of study, types of technology, and advantages and disadvantages. All study designs were included in this explorative review. This leads to the following research questions: [1] What studies are conducted on eHealth technologies used in the treatment of forensic psychiatric patients? [2] Which types of technologies are being researched in the treatment of forensic psychiatric patients? [3] What advantages and disadvantages accompany the different eHealth technologies that are used in the treatment of forensic psychiatric patients?

## Methods

### Inclusion and Exclusion Criteria

Studies that focused on the use of technological interventions to improve the treatment of forensic psychiatric patients were included. The main goal of the technology had to be related to the quality of treatment or the identification of elements essential for treatment, such as criminogenic needs or responsivity. Since the use of technology had to be of added value for the quality of treatment, technologies purely focused on facilitating the diagnostic process, such as computer-assisted testing, were excluded. Furthermore, forensic psychiatric patients had to be the main target group and primary user, so technologies aiming to solely support the work process of therapists were excluded. Finally, technologies that were not related to treatment, but merely focused on court-mandated monitoring of the patient’s location or assessment for court were excluded. Because of the broad scope and exploratory focus of this study, all study designs were included.

### Literature Search

Electronic searches of the databases Scopus, PsycINFO, and Web of Science were conducted in December 2017. An information expert specialized in developing and improving search strategies was involved in the construction of the search strategy. The same search strategy was used in each database. Search terms can be found in Section “Full Electronic Search Strategy” in Appendix. They were divided into two categories: one on treatment within forensic mental healthcare and one on technology. Search terms were identified by studying the search terms of relevant literature, and expert consultation with researchers in forensic mental health. Articles published up until December 2017, written in English, Dutch, or German, were included.

After removing duplicates in EndNote, two authors (Saskia M. Kelders and Hanneke Kip) reviewed the titles. Records were included if titles indicated that the article focused on treatment of forensic psychiatric patients, and if there was a possibility that the study used technology for treatment purposes. Because of the possibility of technology not being mentioned in the title, broad criteria were used to prevent the unjust exclusion of relevant articles. Articles were included if at least one of the authors decided that it was relevant. After screening the titles, obtained abstracts were read by two authors (Yvonne H. A. Bouman and Hanneke Kip), using the aforementioned inclusion and exclusion criteria. If technology was not explicitly mentioned in the abstract, did not contribute to treatment, or had a primary user group that did not consist of patients, records were excluded. Records were included if consensus by both authors was reached. Finally, full-text articles were read by one author (Hanneke Kip). Reasons for excluding and doubts about including articles were discussed with other authors (Saskia M. Kelders and Yvonne H. A. Bouman).

### Data Extraction

The data extraction process of this systematic review was mostly based on the guidelines of the Cochrane Handbook for Systematic Reviews of Interventions (35). However, a quality assessment was not performed because of the heterogeneity of the included study topics and designs: ranging from explorative qualitative studies to RCTs. The data extraction process started with the generation of an elaborate data extraction form, based on the research questions, that was used to standardize the reporting of relevant information from all obtained studies. The data extraction form contained four categories with their own subcategories: type of study, type of technology, and advantages/benefits, disadvantages/barriers. The data extraction form was filled in by one author (Hanneke Kip), a second author (Yvonne H. A. Bouman) was consulted in case of any uncertainties. In the first phase of the data extraction process, all relevant information was copied literally into the narrative data extraction forms. After that, the information in the table was summarized or made more concise. To answer the first research question, study designs were categorized inductively. We distinguished between experimental, quasi-experimental, quantitative cross-sectional, qualitative, and literature studies. A brief summary of the study goal as described in the articles was added as well. We also indicated whether the effectiveness of an eHealth intervention was assessed by a study and, if this was the case, whether it was more effective, less effective or ineffective, based on a classification for defining intervention effectiveness of Morrison et al. (36). According to this classification, interventions can be seen as *more effective* if they led to improvements on the majority of outcomes, were at least as effective as comparison groups and more effective than no intervention groups. Interventions are classified as *less effective* if they led to improvements on a minority of outcome measures or were not as effective as comparison groups, but still more effective than waiting list groups. Interventions classified as *ineffective* did not lead to any improvements. The second research question was answered by coding the studied types of technologies inductively by comparing the nature of the technologies, resulting in six types of technologies. The results table was structured accordingly. For each study, the used technology, its target group, and its goal were described, using the data extraction forms. To answer the third research question, fragments on advantages and disadvantages literally copied from the articles were coded inductively as well. The first author executed the coding process, which included multiple iterations and constant adaptations, until data saturation was reached. During this iterative process, multiple versions of the code schemes were discussed with all authors and adapted accordingly. This resulted in two code schemes with main and subcodes representing different types of main and accompanying specified advantages and disadvantages.

## Results

### Search Results

**Figure 1:**
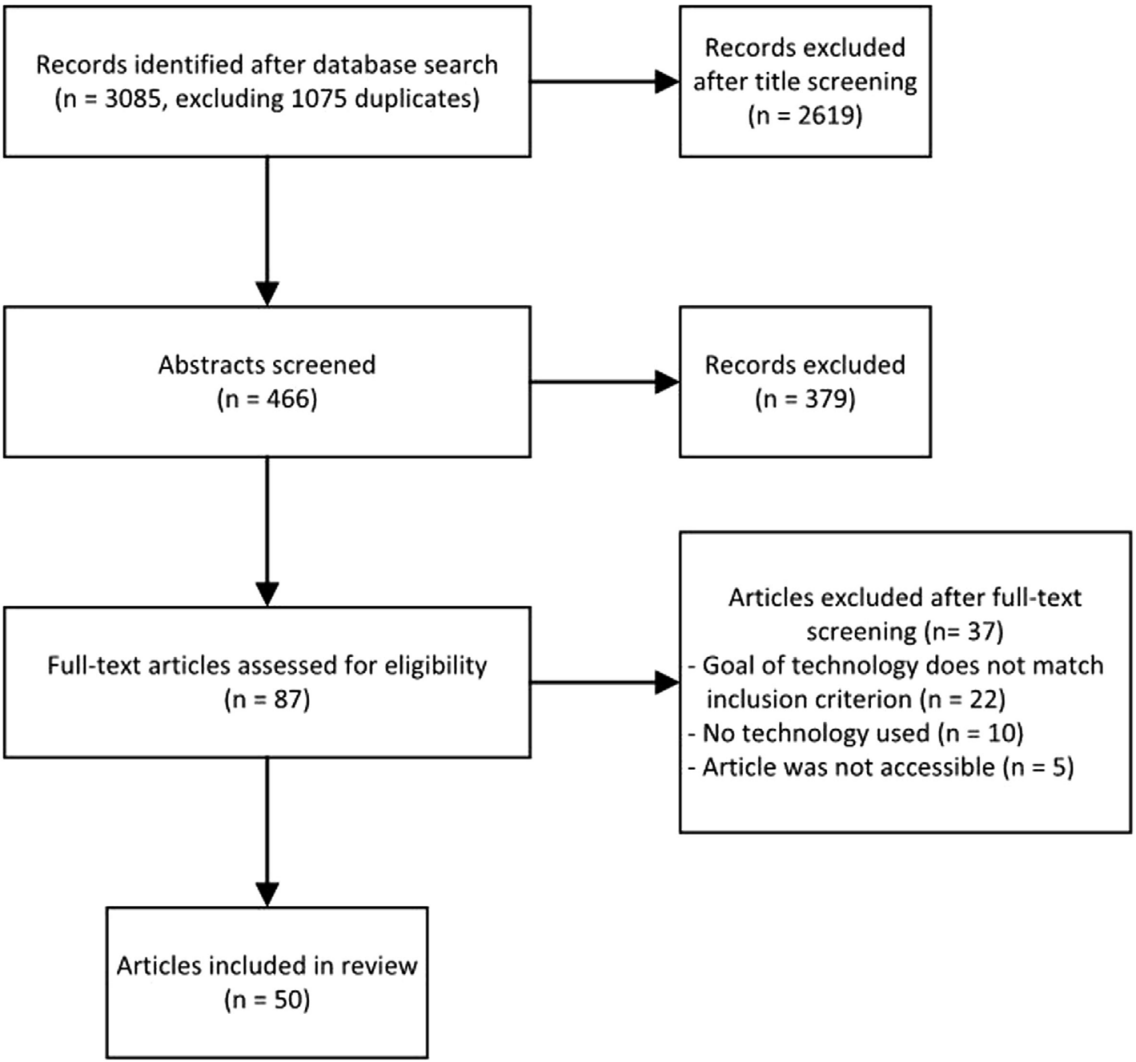
Search strategy and results.

The search strategy, the number of included articles, and reasons for full-text exclusion are provided in Figure 1. The main reason for excluding full-text articles was that they did not match the inclusion criteria: the goal of technology was not directly related to treatment, e.g., the mere monitoring of patients for security purposes, or the target group did not consist of patients, e.g., therapists.

The included 50 studies are provided in Table 1, which is structured based on the research questions. The first column provides the authors and year, the second addresses the first research question by providing the study goal, its design and effectiveness based on the design, the third column describes the technology, and the identified advantages and disadvantages are summarized in the last column. This table serves as the backbone of the result section and can be used to identify references.

**Table 1:** Study characteristics, characteristics of technology, and advantages and disadvantages per study.

Authors, country	Study: Study goal, design, effectiveness	Technology: Technology, target group and goal	Advantages and disadvantages
**Category 1: Interactive, predominantly language-based interventions**			

Berman et al. (37); Sweden	*Goal*: Describing of user’s flow through a hearing voices intervention *Design*: Qualitative study *Effectiveness*: Not assessed	*Technology*: An automated interactive voice response service of the complex telephone-linked care type which conducts automated telephone conversations with patients *Target group*: Forensic psychiatric outpatients and probationers convicted of violent crimes *Goal*: Reducing impulsivity by offering automated psycho-educational interventions based on Dialectical Behavior Therapy, Cognitive Behavior Therapy, and Motivational interviewing	*Advantages*: Intervention accessibility; simulation of situations; potentially effective *Disadvantages*: Difficult to implement

Chaple et al. (38); USA	*Goal*: Evaluating the feasibility of implementing a therapeutic education system (E-TES) in a prison via inmate participation, satisfaction, and skills acquisition *Design*: Experimental study (Stratified Randomized Trial). Control: treatment-as-usual *Effectiveness*: More effective	*Technology*: Computerized intervention: therapeutic education system (E-TES) with interactive multimedia modules *Target group*: Prisoners (male and female) with substance use disorders *Goal*: Learning drug refusal skills, coping with thoughts about using, identifying/managing triggers based on psychosocial treatments	*Advantages*: Patient’s opinion; care in secured settings; lowering threshold; intervention accessibility; tailoring; effective; costs; easy to implement; standardized delivery *Disadvantages*: Patient’s opinion; therapeutic relationship; difficult to implement

Cunningham et al. (39); Canada	*Goal*: Comparing computer- and therapist-delivered interventions in the Emergency Department on feasibility and effectiveness *Design*: Experimental study (three-armed randomized controlled trial). Controls: no intervention or other intervention *Effectiveness*: More effective	*Technology*: Interactive multimedia computer program, viewed on tablet laptops (SafERteens) *Target group*: Adolescents (14–18) reporting alcohol use and violence in the past year *Goal*: Changing attitudes, self-efficacy and readiness to change alcohol use and violence	*Advantages*: Patient’s opinion; fun; tailoring; effective; costs *Disadvantages*: Patient’s opinion

Cunningham et al. (40); Canada	*Goal*: Evaluating the efficacy of behavioral interventions on peer violence and alcohol misuse at 12 months *Design*: Experimental study (three-armed randomized controlled trial). Controls: no intervention or other intervention *Effectiveness*: Ineffective	*Technology*: Interactive multimedia computer program, viewed on tablet laptops (SafERteens) *Target group*: Adolescents (14–18) reporting alcohol use and violence in the past year *Goal*: Changing attitudes, self-efficacy and readiness to change alcohol use and violence	*Advantages*: Effective *Disadvantages*: Therapeutic relationship; Not effective

Elison et al. (41); UK	*Goal*: Exploring Breaking Free Online’s potential to provide support to prisoners’ substance misuse recovery and continuity of care post-release *Design*: Cross-sectional study—quantitative; and qualitative study *Effectiveness*: Not assessed	*Technology*: Computer-assisted therapy intervention with several sessions (breaking free online) *Target group*: Prisoners with substance misuse difficulties *Goal*: Supporting prisoners in strengthening their resilience and build their recovery capital through a range of coping skills, based on cognitive behavior therapy and mindfulness	*Advantages*: Patient’s opinion; lowering threshold; intervention accessibility; potentially effective; time *Disadvantages*: Technological experience; misuse of technology; high costs; difficult to implement

Lee et al. (42); USA	*Goal*: Comparing baseline characteristics and treatment outcomes of forensic patients with participants with no criminal involvement in a psychosocial addiction treatment study *Design*: Experimental study (RCT). Control: treatment-as-usual *Effectiveness*: Ineffective	*Technology*: Web-based substance use intervention (therapeutic education system; E-TES) *Target group*: Forensic outpatients in the first 30 days of their substance abuse treatment program *Goal*: Treating addiction via a psychosocial web-based intervention	*Advantages*: Patient’s opinion; effective *Disadvantages*: Lack of evidence in general

Levesque et al. (27); USA	*Goal*: The development of a stage-based computer-tailored intervention and assessment of its acceptability *Design*: Qualitative study *Effectiveness*: Not assessed	*Technology*: Multimedia computer-tailored intervention (Rise Above Your Situation) *Target group*: Court-mandated juvenile offenders with substance abuse or mental health problems *Goal*: Addressing responsivity by tailoring assessments and guidance to stage of change based on the transtheoretical model of change	*Advantages*: Patient’s opinion; care provider’s opinion; tailoring; potentially effective; costs; time; sensitive information; standardized delivery; behavior change theory

Levesque et al. (27); USA	*Goal*: Examining whether journey to change could improve outcomes of domestic violence treatment *Design*: Experimental study (RCT). Control: treatment-as-usual *Effectiveness*: More effective	*Technology*: Multimedia computer-tailored intervention and print guide (journey to change) *Target group*: Domestic violence offenders *Goal*: Preventing domestic violence perpetration by individualized feedback based on the transtheoretical model of change	*Advantages*: Patient’s opinion; tailoring; effective; costs; time; standardized delivery *Disadvantages*: Not effective

Levesque et al. (43); USA	*Goal*: Examining the opinions of male batterers on an intervention program *Design*: Cross-sectional study—quantitative *Effectiveness*: Not assessed	*Technology*: Expert system: computer programs that mimic the reasoning and problem solving of a human expert *Target group*: Domestic violence offenders in court-mandated programs *Goal*: Activating processes of change in domestic violence offenders based on the transtheoretical model of change	*Advantage*: Patient’s opinion; potentially effective; costs *Disadvantage*: Patient’s opinion

Neville et al. (44); UK	*Goal*: Exploring the existence of relevant violence brief interventions *Design*: Literature study *Effectiveness*: Not assessed	*Technology*: (1) Computerized brief interventions and (2) a touch-screen computer and a video of intimate partner violence (IPV) *Target group*: Young men undergoing treatment for a violent injury *Goal*: Reducing violence	*Advantage*: Fun; time *Disadvantage*: Not effective

Spohr et al. (45); USA	*Goal*: Assessing preferences and evaluating the role of voluntary electronic reminders in achieving early treatment and probation tasks *Design*: Experimental study (three-armed RCT). Controls: other intervention or treatment-as-usual *Effectiveness*: More effective	*Technology*: Web-based intervention with text or email reminders (Motivational Assessment Program to Initiate Treatment; MAPIT) *Target group*: Drug-involved offenders near the start of probation *Goal*: Targeting individual substance use and initiating treatment, based on behavioral theories	*Advantages*: Effective; costs

Tait and Lenton (46); Australia	*Goal*: Systematically reviewing the evidence for the effectiveness of online interventions in reducing sexual violence or IPV *Design*: Literature study *Effectiveness*: Not assessed	*Technology*: Web-based technologies *Target group*: People who perpetrate alcohol-related sexual violence or IPV *Goal*: Reducing sexual violence and alcohol use	*Advantages*: Care in secured settings; lowering threshold *Disadvantages*: Lack of evidence in general

Ranney et al. (47); USA	*Goal*: Examining the secondary effects of a brief alcohol-and-violence ED intervention on depressive symptoms *Design*: Experimental study (three-armed RCT). Controls: other intervention or treatment-as-usual *Effectiveness*: More effective	*Technology*: Interactive multimedia computer program, viewed on tablet laptops (SafERteens) *Target group*: Adolescents (14–18) reporting alcohol use and violence in the past year *Goal*: Changing attitudes, self-efficacy and readiness to change alcohol use and violence	*Advantages*: Effective *Disadvantages*: Not effective

Walters et al. (48); USA	*Goal*: Developing a web-based intervention for substance abusing criminal justice clients *Design*: Qualitative study *Effectiveness*: Not assessed	*Technology*: Web-based, automated intervention on a tablet computer (MAPIT) *Target group*: Criminal justice clients with substance abuse problems, near the start of probation *Goal*: Increasing motivation for substance abuse treatment among clients using illicit substances	*Advantages*: Costs; standardized delivery

Walton et al. (49); USA	*Goal*: Determining the efficacy of brief interventions addressing violence and alcohol use among adolescents in an urban ED *Design*: Experimental study (three-armed RCT). Controls: other intervention or treatment-as-usual *Effectiveness*: More effective	*Technology*: Tablet laptop computer with an interactive animated program with touch screens and audio via headphones (SafERteens) *Target group*: Adolescents reporting past year alcohol use and aggression in the Emergency Department *Goal*: Decreasing the occurrence of peer violence following an ED visit	*Advantages*: Effective; standardized delivery *Disadvantages*: Not effective

Wannachaiyakul et al. (50); Thailand	*Goal*: Investigating the effectiveness of a computerized program for reducing depression among youths with delinquency problems *Design*: Experimental study (RCT). Control: treatment-as-usual *Effectiveness*: More effective	*Technology*: Computerized cognitive-behavioral therapy *Target group*: Youths (14–18 at time of offense) in a juvenile vocational training left *Goal*: Reducing depression among youths in the detention left who have a different context and have limitations accessing traditional CBT	*Advantages*: Fit technological climate; effective *Disadvantages*: Patient’s opinion

Wilson et al. (51); Australia	*Goal*: Exploring whether an online intervention is acceptable, user friendly and contains useful content *Design*: Qualitative; and cross-sectional quantitative (pilot study) *Effectiveness*: Not assessed	*Technology*: Online web-based intervention, accessible via desktop, mobile phone, or tablet *Target group*: First-time convicted drink driving offenders *Goal*: Creating awareness in drink driving and associated alcohol-related behavior to prevent reoffending	*Advantages*: Patient’s opinion; tailoring; costs; behavior change theory *Disadvantages*: Experience with technology

**Category 2: Communication technology for synchronous interpersonal interaction**			

Absalom-Horby et al. (52); UK	*Goal*: Examining the attitudes of staff and relatives of forensic patients toward taking part in an online family intervention *Design*: Cross-sectional—quantitative *Effectiveness*: Not assessed	*Technology*: Video conferencing technology: web camera facilitation for family intervention (e-FFI) *Target group*: Service users of medium secure forensic units with schizophrenia and their family *Goal*: Delivering psychological interventions through the use of Internet technologies such as webcams	*Advantages*: Family’s opinion; geographical barriers *Disadvantages*: Care provider’s opinion; family’s opinion

Absalom-Hornby et al. (53); UK	*Goal*: Describing the implementation a web camera to facilitate a family intervention (e-FFI) in the treatment of schizophrenia *Design*: Cross-sectional—quantitative (*n* = 1 study) *Effectiveness*: Not assessed	*Technology*: Video conferencing technology: web camera facilitation for family intervention (e-FFI) *Target group*: Forensic service users on forensic wards with a diagnosis on schizophrenia spectrum and their families *Goal*: Treating schizophrenia within a forensic service via family interventions	*Advantages*: Family’s opinion; potentially effective; costs

Adjorlolo and Chan (54); China	*Goal*: Providing issues and practice considerations that enhance the results of forensic assessments with video conferencing *Design*: Literature study *Effectiveness*: Not assessed	*Technology*: Video conferencing technology *Target group*: Forensic psychiatric patients and psychologists *Goal*: Obtaining accurate, reliable, and valid assessment results	*Advantages*: Care in secured settings; costs *Disadvantages*: Mental or physical disease; faulty technology; slow connection

Antonacci et al. (55); USA	*Goal*: Reviewing empirical evidence on the use and effectiveness, specifically on forensic psychiatry *Design*: Literature study *Effectiveness*: Not assessed	*Technology*: Video conferencing technology *Target group*: Forensic psychiatric patients and therapists *Goal*: Providing or supporting clinical psychiatric care at a distance	*Advantages*: Patient’s opinion; care in secured settings; effectiveness based on reviews *Disadvantages*: Overhearing; lack of evidence in general

Ax et al. (56); USA	*Goal*: Describing innovations in the assessment and treatment of incarcerated individuals *Design*: Literature study *Effectiveness*: Not assessed	*Technology*: Video conferencing technology *Target group*: Correctional mental healthcare; prisoners *Goal*: Delivering health-care services over a distance between specialty services and non-specialty correction facilities	*Advantages*: Geographical barriers; care in secured settings *Disadvantages*: Lack of evidence in general; high costs

Batastini et al. (57); USA	*Goal*: Providing information on video teleconferencing in forensic and correctional practice *Design*: Literature study *Effectiveness*: Not assessed	*Technology*: Video conferencing technology *Target group*: Forensic psychiatric patients in criminal justice settings *Goal*: Several goals: forensic mental health assessment (e.g., competency determinations, sexually violent predator evaluations), juvenile rehabilitation, group treatment for inmates in segregations	*Advantages*: Patient’s opinion; geographical barriers; care in secured settings; lowering threshold; fit technological climate; effectiveness based on reviews; costs; time *Disadvantages*: Care provider’s opinion; overhearing; therapeutic relationship; lack of evidence in general; faulty technology

Batastini et al. (58); USA	*Goal*: Summarizing all evaluations of telepsychological services that involve videoconferencing equipment in forensic psychiatry *Design*: Literature study (systematic review and meta-analysis) *Effectiveness*: Not assessed	*Technology*: Video conferencing technology *Target group*: Justice-involved substance abusing clients *Goal*: Connect agencies in need of services to agencies that render such services, therefore reducing relapse and recidivism among substance abuse and offender clients	*Advantages*: Patient’s opinion; lowering threshold; effectiveness based on reviews *Disadvantages*: Mental or physical disease; technological experience; detecting subtle behaviors; lack of evidence in general

Brodey et al. (59); USA	*Goal*: Determining the level of satisfaction with telepsychiatry evaluations *Design*: Qualitative study *Effectiveness*: Not assessed	*Technology*: Video conferencing technology *Target group*: Forensic psychiatric patient inmates in a large urban jail *Goal*: Using telepsychiatry for delivering psychiatric services	*Advantages*: Patient’s opinion; geographical barriers; potential effectiveness; time *Disadvantages*: Patient’s opinion

Farabee et al. (60); USA	*Goal*: Comparing the effectiveness of telepsychiatry and in-person treatment-as-usual among parolees *Design*: Quasi-experimental study (randomized field experiment). Control group: treatment-as-usual *Effectiveness*: More effective	*Technology*: Video conferencing technology *Target group*: Parolees from outpatient clinics who received psychiatric care *Goal*: Offering psychiatric treatment via videoconferencing	*Advantages*: Patient’s opinion; effective *Disadvantages*: Therapeutic relationship; faulty technology

Khalifa et al. (61); UK	*Goal*: Literature review on forensic applications of telepsychiatry *Design*: Literature study *Effectiveness*: Not assessed	*Technology*: Video conferencing technology *Target group*: Forensic psychiatric patients and their therapists *Goal*: Delivering mental health services from a distance	*Advantages*: Patient’s opinion; care provider’s opinion; geographical barriers; care in secured settings; effectiveness based on reviews; costs; time *Disadvantages*: Care provider’s opinion; data storage; overhearing; detecting subtle behaviors; lack of evidence in general; high costs; difficult to implement; faulty technology

Manfredi et al. (62); USA	*Goal*: Determining the feasibility of telepsychiatric consultations in an underserved rural jail *Design*: Qualitative study *Effectiveness*: Not assessed	*Technology*: Video conferencing technology *Target group*: Jail inmates who requested or were found to be in need of psychiatric care *Goal*: Increasing access to psychiatric treatment	*Advantages*: Patient’s opinion; care provider’s opinion; care in secured settings; geographical barriers; time *Disadvantages*: Lack of evidence in general

Miller et al. (63); USA	*Goal*: Highlighting the use of teleconferencing for improving access to mental health care for forensic patients *Design*: Literature study *Effectiveness*: Not assessed	*Technology*: Video conferencing technology *Target group*: Forensic clinical practice patients in a child psychiatry outpatient clinic *Goal*: Improving access to services like evaluations, assessment, medication management and treatment coordination	*Advantages*: Patient’s opinion; care provider’s opinion; geographical barriers; costs *Disadvantages*: Data storage; high costs; difficult to implement; no legislation

Miller et al. (64); USA	*Goal*: This study defined telepsychiatry and provided an innovative model of telepsychiatry care delivery in forensic evaluation clinic *Design*: Literature study *Effectiveness*: Not assessed	*Technology*: Video conferencing technology *Target group*: Patients of child and adolescent forensic clinics *Goal*: Assessing and treating forensic psychiatric patients	*Advantages*: Costs; time *Disadvantages*: Care provider’s opinion; data storage; therapeutic relationship; high costs; faulty technology; slow connection; no protocols

Saleem et al. (65); UK	*Goal*: An overview of forensic telepsychiatry in the UK and highlighting practical considerations *Design*: Literature study *Effectiveness*: Not assessed	*Technology*: Videoconferencing technology *Target group*: Community forensic service clients *Goal*: Providing mental health services in a medicolegal context: forensic evaluations, clinical consultation and education	*Advantages*: Costs; time *Disadvantages*: Care provider’s opinion; therapeutic relationship; difficult to implement; no legislation

Sales et al. (66); UK	*Goal*: Literature review on forensic applications of telepsychiatry—update of Khalifa et al. (61) *Design*: Literature study *Effectiveness*: Not assessed	*Technology*: Video conferencing technology *Target group*: Forensic psychiatric patients and their therapists *Goal*: Delivering mental health services from a distance	*Advantages*: Patient’s opinion; geographical barriers; intervention accessibility; costs; time *Disadvantages*: Overhearing; detecting subtle behaviors; lack of evidence in general; costs; implementation; no protocols

Sullivan et al. (67); Australia	*Goal*: Providing an Australian perspective on the use of videoconferencing by forensic mental health services *Design*: Literature study *Effectiveness*: Not assessed	*Technology*: Videoconferencing technology *Target group*: Forensic psychiatric patients *Goal*: Linking remote prisons, courts and psychiatric clinics with distant specialist services, enabling activities including assessment and treatment	*Advantages*: Patient’s opinion; geographical barriers; effectiveness based on reviews; costs; time *Disadvantages*: Overhearing; slow connection; no legislation

Tucker et al. (68); USA	*Goal*: Assessing inmate preferences for telemedicine psychiatric consultation compared to regular care *Design*: Qualitative study *Effectiveness*: Not assessed	*Technology*: Video conferencing technology *Target group*: Inmates who receive psychiatric telemedicine consultations *Goal*: Delivering different kinds of mental services via telemedicine	*Advantages*: Patient’s opinion; time; sensitive information *Disadvantages*: Patient’s opinion

**Category 3: Simulations of offense-related realistic situations**			

Arborelius et al. (69); Sweden	*Goal*: Evaluating of a computer-based system for its effectiveness in distinguishing between offenders and a comparison group *Design*: Cross-sectional study—quantitative *Effectiveness*: Not assessed	*Technology*: Realistically simulated visual events on a computer (reactions on display) *Target group*: Forensic psychiatric patients convicted of violent criminal acts *Goal*: Investigating how offenders understand and interpret social interactions and react to emotions and violence, for both assessment and treatment	*Advantages*: Patient’s opinion; potentially effective

Fromberger et al. (70); Germany	*Goal*: Showing that virtual reality (VR) has an especially high potential for forensic psychiatry *Design*: Literature study *Effectiveness*: Not assessed	*Technology*: VR *Target group*: Forensic psychiatric patients *Goal*: Improving quality of forensic psychiatric care in general	*Advantages*: Care in secured settings; lowering threshold; ecological validity *Disadvantages*: Misuse of technology; lack of evidence in general

Hubal et al. (71); USA	*Goal*: Using embodied conversational agents (ECAs) vignettes for predicting treatment response and misconduct *Design*: Quasi-experimental study (pre-test–post-test). Control: no intervention *Effectiveness*: Ineffective	*Technology*: ECAs: virtual characters rendered on a monitor with whom a user converses *Target group*: Prisoners in correctional institutions *Goal*: Measuring social competency by simulating real interactions with other people; assessing decision-making in a social context through virtual role-playing	*Advantages*: Patient’s opinion; effective; ecological validity *Disadvantages*: Technological experience; overhearing; negative affect; not effective; faulty technology

Montgomery and Brooks (72); USA	*Goal*: Reviewing the progress of incompetent defendants in a program using a television crime-drama “Law and Order” *Design*: Quasi-experimental study (pre- and post-test) *Effectiveness*: More effective	*Technology*: A didactic program, using a popular crime drama series (via TV)—Law and Order *Target group*: Defendants incompetent to stand trial *Goal*: Improving the treatment goal of competency restoration	*Advantages*: Effective

Sygel et al. (73); Sweden	*Goal*: Investigating how male offenders used and reacted to a new interactive computer compared to a control group *Design*: Cross-sectional study—quantitative, and qualitative study *Effectiveness*: Not assessed	*Technology*: Computer-based simulation: a film of an IPV scenario, interactive questions (reactions on display/IPV) *Target group*: Male offenders convicted of IPV toward women *Goal*: Facilitating change in the participant’s violent behavior by allowing him to reflect upon feelings, thoughts and actions during a typical IPV case and practice responses	*Advantages*: Patient’s opinion; potentially effective; sensitive information *Disadvantages*: Negative affect

Wijk et al. (74); Sweden	*Goal*: Developing and pilot testing a simulation system to study and support rehabilitation of mentally disordered offenders *Design*: Qualitative study *Effectiveness*: Not assessed	*Technology*: Computer-based simulation system with videos of a person carrying out everyday activities and decision points (reactions on display) *Target group*: Mentally disordered ward and outpatients who conducted a violent crime, and had a psychotic illness or autistic traits *Goal*: Learning more about patients and identifying dynamic risk factors, and improving rehabilitation	*Advantages*: Patient’s opinion; care provider’s opinion; intervention accessibility; fun; ecological validity

**Category 4: Simulations of realistic offense-related stimuli**			

Benbouriche et al. (75); Canada	*Goal*: Introducing VR applications in the context of forensic psychiatry *Design*: Literature study *Effectiveness*: Not assessed	*Technology*: VR of 3D computer-generated stimuli of children and a virtual character expressing pain *Target group*: Child sexual abusers and violent offenders *Goal*: Measuring deviant sexual interest to predict sexual reoffending and training participants to improve empathic responses	*Advantages*: Effectiveness based on reviews; ecological validity; physiological reactions

Dennis et al. (76); Canada	*Goal*: Determining the perceived age of virtual characters and measuring sexual arousal by using computer-generated images *Design*: Cross-sectional study—quantitative *Effectiveness*: Not assessed	*Technology*: Large screen with virtual characters and penile plethysmography (PPG) to measure sexual arousal *Target group*: Sex offenders *Goal*: Reliably differentiating sex offenders by means of partner receptivity	*Advantages*: Potentially effective; ecological validity; physiological reactions *Disadvantages*: High costs

Renaud et al. (77); Canada	*Goal*: Comparing a VR compared to a standard auditory modality to generate sexual arousal profiles *Design*: Cross-sectional study—quantitative *Effectiveness*: Not assessed	*Technology*: VR with 3D virtual characters depicted naked, PPG *Target group*: Sex offenders, pedophilia *Goal*: Assessing pedophilia and profiles with high ecological validity	*Advantages*: Fun; potentially effective; ecological validity

Renaud et al. (78); Canada	*Goal*: Validating avatars used in the assessment and treatment of deviant sexual preferences *Design*: Cross-sectional study—quantitative *Effectiveness*: Not assessed	*Technology*: VR with avatars, eye-tracking devices and PPG *Target group*: Paraphiliacs with deviant sexual preferences *Goal*: Assessing and treating deviant sexual preferences in a valid way	*Advantages*: Physiological reactions*Disadvantages*: Lack of evidence in general

**Category 5: Games**			

Bacon et al. (79); Australia	*Goal*: Describing the use of the Nintendo Wii Fit in changing engagement in physical activity for patients at risk of obesity *Design*: Cross-sectional study—quantitative; and qualitative study (case studies) *Effectiveness*: Not assessed	*Technology*: Wii Fit: competitive Wii games where bodily movements are required to play them *Target group*: Patients at a secure forensic mental health hospital with a BMI between 25 and 32 *Goal*: Use in rehabilitation to assist in meeting physical activity goals and increasing well-being	*Advantages*: Lowering threshold; fun; potentially effective *Disadvantages*: Difficult to implement

Gooch and Living (80); UK	*Goal*: Comparing findings from videogame research with those among forensic psychiatry *Design*: Literature study *Effectiveness*: Not assessed	*Technology*: Video games: regular, commercial games *Target group*: Forensic clients *Goal*: Supporting the recovery process and serving as a useful relapse prevention strategy by promoting locus of control	*Advantages*: Geographical barriers; care in secured settings; fit technological climate; simulation of situations; fun; effectiveness based on reviews *Disadvantages*: Negative affect; lack of evidence in general

Hodge et al. (81); UK	*Goal*: Testing the feasibility and acceptance of a serious game and describing the development of the prototype game with service users *Design*: Qualitative study *Effectiveness*: Not assessed	*Technology*: Computer-based serious game (StreetWise) *Target group*: Secure forensic mental health service users *Goal*: Supporting and enhancing the rehabilitation of forensic mental health service users prior to their discharge and return to the community	*Advantages*: Patient’s opinion; care provider’s opinion; simulation of situations *Disadvantages*: Care provider’s opinion; technological experience

Reynolds et al. (82); UK	*Goal*: A feasibility study to develop and test the acceptability and usability of a serious game developed with and for service users and providers *Design*: Qualitative study *Effectiveness*: Not assessed	*Technology*: Computer-based serious game (StreetWise) *Target group*: Secure forensic mental health service users *Goal*: Supporting and enhancing the rehabilitation of forensic mental health service users prior to their discharge and return to the community	*Advantages*: Fit technological climate; simulation of situations; fun; potential effectiveness; ecological validity; sensitive information *Disadvantages*: Patient’s opinion; negative affect; therapeutic relationship; misuse of technology; lack of evidence in general; difficult to implement

**Category 6: Platforms with user-generated and shared content**			

Kernsmith and Kernsmith (83); USA	*Goal*: Exploring processes of change and barriers to rehabilitation in an online self-help group for sex offenders *Design*: Qualitative study *Effectiveness*: Not assessed	*Technology*: Website/forum with messages: online self-help group *Target group*: Sex offenders *Goal*: Facilitating a supportive environment but does not providing therapy, based on a cognitive-behavioral model	*Advantages*: Lowering threshold; potentially effective *Disadvantages*: Misuse of technology

Van Gelder et al. (84)Netherlands/USA	*Goal*: Testing whether a future online self reduces delinquent involvement *Design*: Quasi-experimental study (field experiment). Control group: other intervention *Effectiveness*: More effective	*Technology*: Social media with daily messages of a future self (Facebook) *Target group*: Delinquent individuals *Goal*: Reducing delinquent behavior/involvement	*Advantages*: Fit technological climate; effective; time; easy to implement

### Study Designs, Research Goals, and Effectiveness

The included studies were categorized based on their study design (see Table 1). The following categories were identified: experimental studies (*n* = 9), quasi-experimental studies (*n* = 4), qualitative studies (*n* = 12), quantitative cross-sectional studies (*n* = 9), and literature studies (*n* = 16). There was much variation in research goals.

All included *experimental studies* used a randomized controlled trial with either two or three groups. The studies aimed to determine the effectiveness of interventions via measuring treatment outcomes such as depressive complaints, alcohol abuse, and attitudes toward violence. Several studies also paid attention to participation and satisfaction with the intervention. The *quasi-experimental studies* had differing goals: while some determined effectiveness via outcomes such as delinquent involvement, others were more focused on process-related outcomes such as treatment response and progress. Most of the *qualitative studies* investigated the perspective of the patient via measuring outcomes such as use of the technology, patient preferences, acceptability, or satisfaction with the intervention. Some studies also described and analyzed the development process of eHealth interventions. Studies in the *quantitative, cross-sectional* category had an especially broad range of research goals. While some determined attitudes and opinions about interventions, others aimed to design and validated realistic stimuli. Further topics that were studied were feasibility, implementation, or potential effectiveness of interventions. Studies focused not only on ready-to-use interventions but also on interventions that were still being developed. The *literature studies* had in common that they all used scientific literature to provide an overview of the current state of affairs of a specific type of intervention for forensic mental health. However, the included studies ranged from highly structured meta-analyses and systematic reviews to literature reviews and viewpoint papers. Consequently, research goals differed as well: several studies provided a systematic overview of empirical evidence, others provided an overview of existing interventions or technologies, and some focused mainly on the practical applications of technologies in practice.

The effectiveness of an individual intervention was assessed via an experimental or quasi-experimental study by 13 of the 50 studies. Ten of them found that an intervention was as at least as effective as a comparison group and more effective than no intervention groups on most of the outcomes. Three studies found no proof for effectiveness.

### Types of Technology

To provide an oversight of the studied eHealth interventions, we created a categorization of the technologies described in the included articles, based on the way its content was presented and communicated to the user. This resulted in six types of technology.

#### Category 1: Interactive, Predominantly Language-Based Interventions

This type of technology aims to change offense-related cognitions or behavior, mostly via language-based information, assignments, or exercises (*n* = 17). Their content can be delivered via multiple modalities, e.g., written text, videos, or audio, and is often based on theory or existing, evidence-based therapies. The system also reacts on input of the user. Multiple technologies are used in this category, but most included interventions were delivered via a computer or tablet. A broad range of populations was targeted, among others juvenile offenders (27), domestic violence offenders (27, 43), prisoners in general (41), alcohol abusing, violence-involved adolescents (39, 40, 47), and substance abusing prisoners (38). Several web-based interventions were based on theoretical frameworks such as motivational interviewing, the transtheoretical model, cognitive-behavioral therapy or social cognitive theories. These interventions used different ways of delivering content, such as videos, avatars, telephones, tailored written feedback, and emails.

#### Category 2: Communication Technology for Synchronous Interpersonal Interaction

This type refers to the use of technology to enable a patient to directly communicate with a care provider, regardless of location (*n* = 17). The patient always interacts with another human being, and not the technology itself. The main goal of these synchronous communication technologies is to provide an alternative for in-person interaction, so multiple modalities such as sound, video, or text are used. All included studies examined video conferencing technology, which is a two-way interactive video and audio communication system. However, despite using the same technology, these studies did differ in the goal and target group: their focus ranged from, for example, video conferencing in forensic mental health in general (54, 57, 63, 64, 67) to specific target groups, for example, substance abusing patients (58), inmates with psychiatric problems (56, 59, 62, 68), or both schizophrenic patient and family (52, 53).

#### Category 3: Simulations of Offense-Related Realistic Situations

This type of technology focuses on the use of simulations in the treatment of forensic psychiatric patients (*n* = 6). In these simulations, visualized scenarios of possible events are presented to patients. The goal of this type of technology is to explore attitudes or behavioral responses to offense-related situations that are viewed as realistic and personally relevant by the user. Some studies used videos with real actors and authentic situations in which the user had to decide on behavioral reactions (69, 73, 74), while others simulated situations via virtual embodied conversational agents to conduct a dialog with prisoners (71), existing popular crime drama series (72), or VR (70).

#### Category 4: Simulations of Offense-Related Realistic Stimuli

This type of technology presented realistic depictions of stimuli related to the offense of forensic psychiatric patients (*n* = 4). The goal of using these stimuli is to elicit behavioral, emotional, cognitive, and/or physiological responses of the patient who are relevant for treatment. These stimuli are not interactive, so they do not respond to the actions of the patient, and are not situations. All included studies primarily focused on sex offenders by presenting computer-animated virtual characters depicting realistic naked human beings of several age categories and genders, via either VR or large screens (75–78).

#### Category 5: Games

This type of technology entails both the usage of existing, commercial games for treatment purposes, and serious games developed specifically for the treatment of forensic psychiatric patients (*n* = 4). Game elements are always present: the user has to improve his own achievement, or is competing with other users. Studies examined existing, commercial games to improve the recovery process or meet physical activity goals (79, 80), but a serious game specifically developed for rehabilitation purposes was described as well (81).

#### Category 6: Platforms with User-Generated and Shared Content

This sixth type refers to technologies in which patients can create and react on content, and add and share existing content (*n* = 2). These platforms can be freely accessible to anyone, e.g., social media, or an approved account can be required, e.g., private forums. The goal of these platforms is for users to share and read material like experiences or opinions to support them in refraining from delinquent behavior. Two studies focused on these kinds of platforms: one was a web-based self-help group for sex offenders (83), the other one made use of social media to reduce delinquent behavior (84).

### Advantages

Types of advantages and disadvantages of eHealth technologies were extracted from the included studies via inductive coding. We identified main codes and accompanying subcodes based on relevant fragments from the article, which are provided in Table 2. The subcodes that were found in each article can be found in Table 1.

**Table 2:** Main and specified advantages of eHealth and amount of studies they were mentioned by (*n*).

Main advantage	Specifications	*n*
**Advantages for individuals**		
Positive opinion	Opinion of forensic psychiatric patients	26
	Opinion of providers of care	7
	Opinion of family of patient	2
Increasing patients’ access to care	Overcoming geographical barriers	10
	Receiving care in highly secured settings	10
	Lower perceived threshold to participate	8
	Intervention accessible anywhere, anytime	5
Fit with patient’s needs, preferences, and living environment	Fun to use	7
	Fit with current technological climate	5
	Simulation of situations perceived as realistic	4
	Tailored to specific characteristics of patients	5

**Advantages for forensic mental health**		
Effectiveness	Potential effectiveness	13
	Effectiveness based on experimental designs	13
	Effectiveness based on reviews	7
Efficiency	Cost savings	17
	Time savings	14
	Easy to implement in practice	2
Unique information	Situations with high ecological validity	7
	Eliciting more sensitive information	4
	Measuring physiological or unconscious reactions	3
Fidelity in delivering interventions	Delivering content and structure in a standardized way	5
	Behavior change theory	2

Several types of advantages were relevant for individual people who are in contact with the technology. Multiple articles mentioned the *positive opinion* about an eHealth intervention of people who were using or in direct contact with it. This opinion could entail positive attitudes, a high satisfaction or high acceptance of an eHealth intervention before, during or after its use. Studies also indicated that technology can *increase patients’ access to care* which makes it easier for them to receive treatment. This can be related to overcoming actual physical barriers to receiving care such as traveling distance, or access to care in highly secured settings. The subjective threshold to following treatment can be influenced by technology as well, for example, via privacy and anonymity, or the possibility of accessing an intervention from home and 24/7. Technology also offers new opportunities to involve loved ones in treatment. Another advantage is that technology can closely *fit patients’ needs, preferences, and living environment*. Patients can find specific technologies fun to use, e.g., serious games or VR, or technology can be developed in such a way that it is automatically tailored to specific characteristics. Also, compared with in-person treatment, technology is better able to create situations that are perceived as realistic and personally meaningful by patients.

Besides individual advantages, studies also described the added value that technology can have for forensic mental health. An important advantage is that eHealth interventions can be as *effective* as or even more effective than care as usual in reaching their intended goals. Included studies of a more observational nature indicated that the intervention they studied had a lot of potential to be effective, but no definite conclusions could be drawn based on their preliminary qualitative or quantitative results. The included experimental and quasi-experimental studies were able to provide some more insight into effectiveness, and reported mainly positive effects, as can be seen in Table 1. eHealth interventions can also increase the *efficiency* of forensic mental health care, which refers to practical advantages for forensic settings. Time and costs were claimed to be saved, for example, because eHealth can take away some of the work of therapists, an eHealth intervention can be quicker than in-person care, and many eHealth interventions are easily scalable, so multiple patients are able to follow an intervention at the same time without an increase of labor of care providers or a decrease in safety of patient, provider, or society. However, none of the studies that made statements about reduced costs and time conducted a systematic cost-effectiveness analysis. Studies also stated that eHealth technology can provide *unique information* that cannot or is difficult to elicit via in-person interventions. Technology was said to create situations with a high ecological validity, in which behavioral reactions can be observed and trained as they occur. Furthermore, it can be used to collect non-verbal information about physiological or unconscious reactions via the use of, e.g., biofeedback, eye-tracking, or measuring sexual arousal. A final advantage is related to *fidelity*: via technology, interventions can be delivered exactly as was intended, without error or unintended deviations from the desired situation. The standard procedures, structure, content, and evidence-based methods of an intervention can be delivered to patients in a standardized way, meaning that every patient gets the same treatment.

### Disadvantages

Besides advantages, disadvantages were identified as well. They are mentioned and explained in the Table 3 and the accompanying text below.

**Table 3:** Main and specified disadvantages of eHealth and amount of studies they were mentioned by (*n*).

Main disadvantage	Specification	*n*
**Disadvantages for individuals**		
Negative opinion	Opinion of forensic psychiatric patients	7
	Opinion of providers of care	6
	Opinion of family of patient	1
Not suitable for every patient	Experience with technology	5
	Mental or physical diseases	2
Privacy	Patients can be overheard	6
	Data not stored securely	3
Decrease or lack in-person contact	Negative influence on therapeutic relationship	7
	Not detecting subtle behaviors or signs	3
Adverse negative consequences	Patients’ misuse of technology	4
	Excessive experience of negative affect	4

**Disadvantages for forensic mental health**		
Effectiveness	Not enough evidence in general	13
	Single intervention is not effective	7
Inefficiency	Difficult to implement in practice	9
	High costs	7
Technological malfunctioning	Faulty technology	6
	Slow or lost connection	3
Lack of standardization	No clear protocols, guidelines, or standards	2
	No national legislation	2

Disadvantages for individual people were identified in the included studies. First of all, *negative opinions* of people directly involved with an eHealth intervention were reported. Studies found that attitudes about the technology were negative, or that the acceptance of a technology was low. It was also mentioned that not all technologies can be used by *every type of patient*, for example, if they have hallucinations or are physically incapable of using the system. Patients might also have low eHealth literacy because they lack knowledge and skills to use specific technologies, or they might be used to commercial products and are underwhelmed by an eHealth intervention. Another issue for individual patients is related to *privacy* and the confidentiality of sensitive information. Insecurely stored data can be accessed by unauthorized parties, or, especially in the case of communication technologies, patients might be overheard by staff or other patients. Furthermore, it was proposed that a *decrease or lack of in-person contact* between patient and therapist when using technology could have a negative impact on the therapeutic relationship, since both parties might perceive a greater emotional distance. Communication via technology can also make it harder or impossible to detect subtle but relevant behaviors or other signs such as fidgeting under the table or smell. eHealth interventions can also give rise to some *adverse negative consequences*: patients might misuse the technology, for example by using VR stimuli of children to arouse themselves, or technology can arouse excessive, unwanted negative affect in a patient.

eHealth can also have disadvantages for the domain of forensic mental health. The section on advantages already provided insight into the *effectiveness* of eHealth. However, studies also mentioned the lack of evidence of effectiveness, either for an entire domain, one intervention, or a specific outcome. Reviews often indicated that there is not enough evidence for a type of eHealth technology, for example, teleconferencing or VR. It was said that too little studies were conducted to make statements about their effectiveness. Besides these more general statements, some experimental studies also found that a single eHealth intervention was not effective in general, or on specific outcome measures. eHealth can also be *inefficient* when its development, implementation, or long-term use have practical negative consequences for an organization. Development, start-up costs, and maintenance of technology were often said to be costly, and insurance companies did not cover these costs. Also, several studies indicated that implementation in practice is difficult because of multiple reasons, such as unawareness of the existence of eHealth by stakeholders, too much reliance on the time and efforts of staff, or lack of physical space to set up a system. Furthermore, *technological malfunctioning* can negatively affect the quality of an eHealth intervention: software might contain bugs, equipment can fail, it can be too outdated to use, or the connection might be lost or be too slow. Finally, an observed disadvantage for eHealth is the *lack of standardization*: clear protocols, guidelines, and national legislation to optimize and standardize the use of eHealth in practice are insufficient or non-existent.

### Combination of Results

Table 4 combines the results provided in Table 1 to create an overview of the study design, effectiveness, advantages, and disadvantages for each of the six identified types of technology.

**Table 4:** Study design, effectiveness, advantages, and disadvantages categorized per type of technology.

	Study design	Effectiveness	Advantages	Disadvantages
Category 1: Interactive, predominantly language-based interventions (*n* = 17)	Experimental: *n* = 9 Qualitative: *n* = 4 Quantitative cross-sectional: *n* = 2 Literature study: *n* = 2	More effective: *n* = 7 Ineffective: *n* = 2 Not assessed: *n* = 8	Effectiveness: *n* = 13 Efficiency: *n* = 10 Positive opinion: *n* = 8 Fit with patient’s needs, preferences and environment: *n* = 8 Fidelity in delivering interventions: *n* = 5 Increasing patient’s access to care: *n* = 4 Unique information: *n* = 1	Effectiveness: *n* = 7 Negative opinion: *n* = 4 Inefficiency: *n* = 3 Not suitable for every patient: *n* = 2 Decrease or lack of in-person contact: *n* = 2 Adverse negative consequences: *n* = 1

Category 2: Communication technology for synchronous interpersonal interaction (*n* = 17)	Literature study: *n* = 11 Qualitative: *n* = 3 Quantitative cross-sectional: *n* = 2 Quasi-experimental: *n* = 1	More effective: *n* = 1 Not assessed: *n* = 16	Positive opinion: *n* = 13 Increasing patient’s access to care: *n* = 12 Efficiency: *n* = 12 Effectiveness: *n* = 9 Fit with patient’s needs, preferences and environment: *n* = 1 Unique information: *n* = 1	Negative opinion: *n* = 7 Privacy: *n* = 7 Decrease or lack of in-person contact: *n* = 7 Effectiveness: *n* = 7 Technological malfunctioning: *n* = 6 Inefficiency: *n* = 6 Lack of standardization: *n* = 5 Not suitable for every patient: *n* = 2

Category 3: Simulations of offense-related realistic situations (*n* = 6)	Quasi-experimental: *n* = 2 Quantitative cross-sectional: *n* = 2 Qualitative: *n* = 1 Literature study: *n* = 1	More effective: *n* = 1 Ineffective: *n* = 1 Not assessed: *n* = 4	Positive opinion: *n* = 4 Effectiveness: *n* = 4 Unique information: *n* = 4 Increasing patient’s access to care: *n* = 2 Fit with patient’s needs, preferences and environment: *n* = 1	Adverse negative consequences: *n* = 3 Effectiveness: *n* = 2 Not suitable for every patient: *n* = 1 Privacy: *n* = 1 Technological malfunctioning: *n* = 1

Category 4: Simulations of offense-related realistic stimuli (*n* = 4)	Quantitative cross-sectional: *n* = 3 Literature study: *n* = 1	Not assessed: *n* = 4	Unique information: *n* = 4 Effectiveness: *n* = 3 Fit with patient’s needs, preferences and environment: *n* = 1	Effectiveness: *n* = 1 Inefficiency: *n* = 1

Category 5: Games (*n* = 4)	Qualitative: *n* = 2 Quantitative cross-sectional: *n* = 1 Literature study: *n* = 1	Not assessed: *n* = 4	Fit with patient’s needs, preferences and environment: *n* = 4 Effectiveness: *n* = 3 Increasing patient’s access to care: *n* = 2 Positive opinion: *n* = 1 Unique information: *n* = 1	Negative opinion: *n* = 2 Adverse negative consequences: *n* = 2 Effectiveness: *n* = 2 Inefficiency: *n* = 2 Not suitable for every patient: *n* = 1 Decrease or lack of in-person contact: *n* = 1

Category 6: Platforms with user-generated and shared content (*n* = 2)	Quasi-experimental: *n* = 1 Qualitative: *n* = 1	More effective: *n* = 1 Not assessed: *n* = 1	Effectiveness: *n* = 2 Increasing patient’s access to care: *n* = 1 Fit with patient’s needs, preferences and environment: *n* = 1 Efficiency: *n* = 1	Adverse negative consequences: *n* = 1

The table shows that all experimental studies were conducted on interactive, language-based interventions, and most of these studies found that an eHealth intervention is as effective as or even more effective than a control intervention. Effectiveness was found most as both an advantage and disadvantage, and fidelity in delivering content and structure is mentioned only by these studies. The second category on teleconferencing contains almost all literature studies. These reviews mostly include studies on telepsychiatry in general, whereby the authors related their findings to forensic psychiatry. Opinions of individuals were found most in this category, as both an advantage and disadvantage. Compared with other categories, a lot of these studies paid attention to the increased access to care for patients. Technologies that simulate situations were studied in various ways. Overall, positive opinions and the unique possibilities of these interactive technologies were identified more often than in most other categories, just as the adverse negative consequences of a technology. No studies on effectiveness were conducted on technologies that simulated realistic stimuli, the fourth category. The possibility of technology to create and acquire unique information was acknowledged by all four included studies. The fifth category, games, also contains no studies on effectiveness. Most advantages and disadvantages were related to the individual patient, with all studies mentioning the advantage of the fit between the patient and the technology. The final category, platforms with user-generated content, comprises only two studies, of which one found evidence for effectiveness.

When looking at all mentioned advantages, effectiveness was stated in studies on all categories, just as the fit of the technology with the patient. However, efficiency was not identified in studies on technologies that simulated situations and stimuli, while the majority of these studies mentioned unique information as an advantage. Overall, less disadvantages then advantages were identified, so most of the disadvantages were stated only once or twice per type of technology. Effectiveness and adverse negative consequences are present in five categories, most other disadvantages are found in less categories. Finally, most of the mentioned disadvantages are complementary to the advantages, for example, both positive and negative opinions were identified in studies on teleconferencing, indicating that this is an important topic for studies on these types of technology.

## Discussion

This systematic review provided an overview of the research on eHealth technologies that are used in the treatment of forensic psychiatric patients. The 50 included studies showed a broad range of eHealth technologies that were studied using different research methods, ranging from RCTs to exploratory qualitative studies. Most studies on effectiveness were conducted on language-based interventions, while most exploratory studies focused on technologies that provided an experience and made less use of language. Despite these differences, many publications mentioned the same type of advantages. The opinions of patients and therapists were positive, access to care was increased, the technology fitted the patient, interventions were—or were expected to be—effective and efficient, technology was said to increase fidelity of treatment, and offered new possibilities and information. Disadvantages were that not everyone was enthusiastic about and able to use technology, there were concerns about privacy, in-person contact could decrease, technology could have unintended negative consequences, not every study found strong proof of effectiveness and efficiency, technology could contain errors, and many settings did not have regulations or protocols for eHealth. When comparing the advantages to the disadvantages, it becomes clear that there is a lot of potential and much has been achieved at this point in time, but there are also many opportunities that are not used.

Important advantages of technology were related to technology being able to deal with the complex nature of the forensic psychiatric population. Technology can take the low literacy and education level of forensic psychiatric patients into account (15) by not relying primarily on language and cognitive reflection; it can create real-life, interactive situations in which skills can be trained (56, 70, 74, 77), or information on reactions can be gathered via physiological measures which can be integrated in treatment (27). However, most of these types of technologies are not thoroughly studied, so more studies on technologies such as VR or wearables that monitor arousal are required to determine whether they actually have added value for forensic psychiatric patients.

When looking at the use of technology in practice, many studies reported positive attitudes of both patients and care providers. A positive attitude increases the motivation to actually use a technology in the intended way (30). However, none of the studies paid attention to matters related to the use of technology such as engagement and adherence, despite the knowledge that treatment motivation and completion is low in forensic psychiatric patients (10). Research into the manner in which technology is used by patients and suitable methods to increase their engagement and adherence is required to gain more insight into how and why technology can motivate forensic patients. A way to increase adherence is via persuasive design (33, 34), which was recommended by one of the included studies (48). A specific persuasive element that multiple studies did mention as an advantage is tailoring (18, 27, 38, 40, 58). Tailoring has been recommended as a way to overcome the current predominant “one-size-fits-all” approach in forensic mental health by increasing the fit between the technology and the user (19, 51). Research has shown that tailoring is of added value for eHealth in general [e.g. Ref. (22)], but most interventions for forensic mental health did not use this specific possibility. To conclude, more research needs to be conducted on the interrelationships between technology and treatment motivation, and ways to increase engagement and adherence such as tailoring need to be identified.

Most included experimental and quasi-experimental studies found promising results for web-based interventions. In the majority of the included studies, web-based interventions were as effective as and, in some cases, more effective than in-person interventions. However, many technologies were not studied as extensively as this category, so to create a thorough evidence base for all categories, more evaluation studies are recommended (58, 60, 76). A more specific recommendation provided by several studies was to determine what types of interventions work best for which type of patients, and which mechanisms of change contribute to these differences between individuals (47, 48, 59, 76, 83). Insight in these mechanisms enables better tailoring of interventions to specific groups of patients. These kinds of recommendations are in line with recent visions on eHealth evaluations in general (85, 86), which stress the importance of determining what works best for whom to gain more insight into the working mechanisms.

A final advantage that was mentioned by several included studies is the possibility to incorporate existing guidelines and treatment approaches in eHealth interventions. Blended care, a format in which the use of eHealth is combined and integrated with in-person care, is an especially promising possibility (20). However, only one included study recommended that it should be examined how to best implement computer-delivered interventions in real world settings (48). Most studies did not pay attention to the implementation and integration of eHealth in existing care pathways. For eHealth to be as effective and efficient as possible, it needs to fit the context in which it is used seamlessly: technology should not be used as a separate, standalone tool, but has to be embedded within the current situation (87), among other things via integration in evidence-based treatment approaches such as cognitive behavior therapy and the risk-need-responsivity model. Especially studies on telepsychiatry stressed the need for protocols, standards, and guidelines to achieve this.

Based on the results of this review, it becomes clear that an issue important for eHealth in general also applies to forensic mental health: despite promising studies that show potential, there still is a large gap between potential and current practice, and most interventions fail to have actual clinical benefit in real world settings (86). To bridge this gap and achieve eHealth’s potential, it is essential to create a good fit between the technology, the people involved, and the existing context with its treatments approaches or interventions.

A way to increase the fit between technology, people, and their context is by conducting a good development process (88, 89). Not many of the included studies discussed the development of their interventions. The ones who did discuss it pointed out the importance of iterative development with continuous evaluation cycles (37, 53, 59) and the incorporation of opinions, preferences, and characteristics of people (37, 54, 57, 71, 81, 82). This is in line with recent insights into eHealth development, which state that a good development process requires iterative, evidence-based strategies that acknowledge the complex interrelations between people, technology, and the health-care context (85, 86). These strategies should be derived from multiple disciplines, such as persuasive design, human-centered design, participatory development, business modeling, engineering, and psychology (87). Interventions should not be developed in an expert-driven, non-iterative way (86), but this seemed to be the case for most of the included interventions. Also, due to a virtual absence of insight into the development process, little knowledge on the most optimal way to develop eHealth that fits with forensic mental health practice is present. Consequently, more studies on eHealth in forensic mental health should apply, describe, and critically evaluate development methods.

Implementation was another essential activity to which not much studies paid attention, despite the fact that research and practice have shown that it is a very important yet difficult endeavor (90). Some studies made recommendations for the implementation: they mentioned the necessity of accounting for resistance by patients and therapists, creating an infrastructure for dissemination, and financing (27, 42, 48, 53, 55). The importance of these kinds of activities has indeed been acknowledged by other studies and can be accounted for via using approaches such as business modeling (91) and participatory development (92).

### Strengths and Limitations

The main strength of this study is that it used a systematic approach based on the Cochrane guidelines to provide a broad and extensive overview of the current state of research on eHealth interventions in forensic mental health. Despite the thorough execution of this review, it has several limitations. First of all, time between the development and evaluation of a technology and the publication of a study might take up to a couple of years (85), which causes that the most recent interventions and studies that are being conducted at the moment could not be accounted for in this review. Furthermore, because the goal of this systematic review was to provide an overview of the current state of affairs of research, all available studies were included, regardless of their quality and type of results. Consequently, not all results of included studies might be valid or reliable and thus it cannot be guaranteed that all advantages and disadvantages mentioned by these studies are factual and objective. Some advantages and disadvantages mentioned in the articles were based on qualitative results or non-systematic observations combined with reasoning based on existing literature. Again, more research is required to determine whether they can be objectively observed in forensic mental health care. We recommend that a systematic review specifically focused on effectiveness is executed in the near future, when more experimental studies on this increasingly studied topic have been published.

## Conclusion

Based on the results of this review, we conclude that eHealth has many actual and potential advantages for forensic mental health. Some especially promising advantages are tailoring, effectiveness—which was mostly examined in web-based interventions—and, specifically relevant for non-language-based technologies, the acquisition of unique information via the use of technology. However, most interventions did not yet fully benefit from the possibilities of the different types of available technologies. To take eHealth in forensic mental health to the next level, it is important to ensure that the use of technology has actual added value for the patient and treatment. eHealth technology needs to be integrated in in-person treatment instead of using it as a separate addition to care, and it needs to closely fit the needs and preferences of both patients and therapists. Consequently, to achieve the benefits and overcome the barriers, eHealth should be developed in such a way that there is a good fit between technology, people, and the context.

## Author Contributions

HK, YB, and SK designed the study and wrote the protocol. HK conducted literature searches. HK and SK screened the titles, while HK and YV screened the abstracts and analyzed the data. HK wrote the first draft of the manuscript and YB, SK, and LG-P contributed to and have approved the final manuscript.

## Conflict of Interest Statement

All authors declare that they have no conflicts of interest. The reviewer ND and handling editor declared their shared affiliation.

## Appendix

### A. Full Electronic Search Strategy

*Search string #1 on treatments in forensic mental health settings*

forensic
violen*
crim*
delinquen*
offend*
maximum secur*
W/3
treatment*
psychiatr*
patient*
setting*
assessment*
diagno*
rehabilitation
parole
probation
therap*
coach*
intervention*

*Search string #2 on technology*

E-Health
eHealth
M-Health
mHealth
technolog*
device*
platform*
videoc*
tele*
mobile
*phone
SMS;
web*
“virtual Reality”
virtual
“augmented reality”
wearable*
smart*watch*
game*
online
computer
